# In Silico Structure-Based Design of Antiviral Peptides Targeting the Severe Fever with Thrombocytopenia Syndrome Virus Glycoprotein Gn

**DOI:** 10.3390/v13102047

**Published:** 2021-10-11

**Authors:** Shuo-Feng Yuan, Lei Wen, Kenn Ka-Heng Chik, Jiang Du, Zi-Wei Ye, Jian-Li Cao, Kai-Ming Tang, Rong-Hui Liang, Jian-Piao Cai, Cui-Ting Luo, Fei-Fei Yin, Gang Lu, Hin Chu, Mi-Fang Liang, Dong-Yan Jin, Kwok-Yung Yuen, Jasper Fuk-Woo Chan

**Affiliations:** 1 State Key Laboratory of Emerging Infectious Diseases, Carol Yu Centre for Infection, Department of Microbiology, Li Ka Shing Faculty of Medicine, The University of Hong Kong, Pokfulam, Hong Kong, China; yuansf@hku.hk (S.-F.Y.); wenlei07@hku.hk (L.W.); kchik929@connect.hku.hk (K.K.-H.C.); zwye@hku.hk (Z.-W.Y.); caojenny@connect.hku.hk (J.-L.C.); kmtang20@hku.hk (K.-M.T.); liangrh@hku.hk (R.-H.L.); caijuice@hku.hk (J.-P.C.); cuiting@hku.hk (C.-T.L.); hinchu@hku.hk (H.C.); kyyuen@hku.hk (K.-Y.Y.); 2 Key Laboratory of Tropical Translational Medicine of Ministry of Education, Hainan Medical University, Haikou 571199, China; dujiang@hainmc.edu.cn (J.D.); yinfeifei@hainmc.edu.cn (F.-F.Y.); hy0211004@hainmc.edu.cn (G.L.); 3 Academician Workstation of Hainan Province, Hainan Medical University, Haikou 571199, China; 4 Hainan Medical University-The University of Hong Kong Joint Laboratory of Tropical Infectious Diseases, Hainan Medical University, Haikou 571101, China; 5 Hainan Medical University-The University of Hong Kong Joint Laboratory of Tropical Infectious Diseases, The University of Hong Kong, Pokfulam, Hong Kong, China; 6 Key Laboratory for Medical Virology and National Institute for Viral Disease Control and Prevention, Chinese Centre for Disease Control and Prevention, Beijing 102206, China; mifangl@vip.sina.com; 7 School of Biomedical Sciences, Li Ka Shing Faculty of Medicine, The University of Hong Kong, Pokfulam, Hong Kong, China; dyjin@hku.hk

**Keywords:** antiviral, bunyavirales, peptide, SFSTV, tick, treatment

## Abstract

Severe fever with thrombocytopenia syndrome virus (SFTSV) is an emerging tick-borne bunyavirus in Asia that causes severe disease. Despite its clinical importance, treatment options for SFTSV infection remains limited. The SFTSV glycoprotein Gn plays a major role in mediating virus entry into host cells and is therefore a potential antiviral target. In this study, we employed an in silico structure-based strategy to design novel cyclic antiviral peptides that target the SFTSV glycoprotein Gn. Among the cyclic peptides, HKU-P1 potently neutralizes the SFTSV virion. Combinatorial treatment with HKU-P1 and the broad-spectrum viral RNA-dependent RNA polymerase inhibitor favipiravir exhibited synergistic antiviral effects in vitro. The in silico peptide design platform in this study may facilitate the generation of novel antiviral peptides for other emerging viruses.

## 1. Introduction

Members of the order *Bunyavirales* are a group of enveloped RNA viruses with segmented, single-stranded, negative-sense or ambisense RNA genomes [1]. The majority of human-pathogenic members of *Bunyavirales* are transmitted by arthropod vectors such as ticks, mosquitoes, and sandflies, with the exception of hantaviruses and arenaviruses which are usually transmitted through contact with infectious rodent excreta. Bunyaviruses can cause a range of human diseases such as acute self-limiting febrile illness, encephalitis, and viral hemorrhagic fever. Bunyaviruses that cause life-threatening viral hemorrhagic fever include severe fever with thrombocytopenia syndrome virus (SFTSV), Rift Valley fever virus (RVFV), Crimean-Congo hemorrhagic fever virus (CCHFV), and hantaviruses. SFTSV is an emerging tick-borne virus in the genus *Bandavirus* in the family *Phenuiviridae*, order *Bunyaviraless* [2]. The genome of SFTSV consists of three RNA segments, namely, the large (L), medium (M), and small (S) segments [3]. The L segment encodes the RNA-dependent RNA polymerase (RdRp), which mediates viral RNA replication and synthesis. The M segment encodes the viral envelope glycoproteins, glycoprotein N (Gn), and glycoprotein C (Gc), which mediate fusion between the viral and host cell membranes. The S segment encodes the viral nucleoprotein (NP) and the nonstructural protein (NSs). NP is the most abundantly expressed protein in SFTSV viral particles and infected cells. NSs is a potentially important virulence factor of SFTSV that inhibits the host innate antiviral response [4]. The C-type lectin, DC-SIGN, has been identified as one of the factors for SFTSV attachment and entry. In particular, SFTSV Gn/Gc pseudotypes infect human lung (BEAS-2B, A549, and H1299), kidney (293T), liver (HepG2), colon (Caco-2), retinal epithelium (RPE), and glioblastoma (U373) cell lines, as well as human monocyte-derived dendritic cells, with high efficiency [5]. Another report showed that glucosylceramide, the glucose-modified lipid, is required for efficient SFTSV entry into human bone osteosarcoma epithelial (U2OS) cells [6].

SFTSV was first discovered from patients in the Henan Province of China, who presented with fever, thrombocytopenia, leukopenia, and multiorgan dysfunction syndrome (termed severe fever with thrombocytopenia syndrome, SFTS) [7]. More recently, SFTSV has subsequently also been isolated from infected humans, ticks, and/or mammals in Japan, South Korea, and Vietnam, indicating that the geographical distribution of the virus has likely been underestimated in the past [8,9,10,11]. SFTS is characterized by acute fever, thrombocytopenia, and hemorrhagic complications, with a mortality rate of up to about 10–20% [7,12,13]. Other systemic manifestations such as lymphadenopathy, hepatosplenomegaly, gastrointestinal symptoms, proteinuria, and coma may also occur. Despite its clinical significance, effective antivirals for SFTS remains limited. We have previously conducted a drug repurposing program by establishing a two-tier test system to rapidly screen a Food and Drug Administration (FDA)-approved drug library for drug compounds with anti-SFTSV activity [14]. We identified a number of drug compounds that inhibited SFTSV replication at low micromolar concentrations, including hexachlorophene, which likely targets the SFTSV Gc glycoprotein [14]. To find more candidate antivirals expand, in this study, we designed a series of cyclic peptides based on the interacting hotspots of the other SFTSV envelop glycoprotein, the Gn glycoprotein. Viral neutralization sites are reported to locate on both the Gn and Gc domains of other phleboviruses. In one of the studies, five linear epitopes were identified using rabbit pAb α-SGn. The epitopes comprised are listed as E1 (^196^FSQSEFPD^203^), E2 (^232^GHSHKII^238^), and E5 (^316^SYGGM^320^) on domain II and E3 (^256^VCYKEGTGPC^265^) and E4 (^285^FCKVAG^290^) on domain III of SFTSV-Gn [15]. In another study, monoclonal antibody mAb4-5 and mAb 10 bind to domain III and II, respectively, indicating that these subdomains were highly antigenic regions on the SFTSV-Gn [16]. Here, we identified a potent peptide, designated HKU-P1, that not only neutralizes SFTSV but also exhibits antiviral synergy with the broad-spectrum antiviral favipiravir which has previously been reported to exhibit anti-SFTSV effect [17,18].

## 2. Materials and Methods

### 2.1. Design of Cyclic Peptides Based on the Interacting Hotspots

The crystal structure of SFTSV Gn and MAb 4-5 (code: 5Y11) was downloaded from the Protein Data Bank (PDB) [16,19]. Binding free energy (interface score) of the complex was analyzed with Rosetta InterfaceAnalyzer [20]. The interacting hotspots in Gn-mAb4-5 interface were identified by using Rosetta Residue Energy Breakdown protocol [21]. The binding energy decomposition of mAb4-5 residues within 4.0Å of SFTSV Gn were calculated. The linear pentapeptide 100–104 (LYS-ARG-ARG-GLY-PHE) was extended for cyclization due to the higher binding affinity. Two to three residues were inserted into the gap to close the conformation by forming an isopeptide bond with residue 102 sidechain. The arginine in position 102 was substituted to lysine for better synthesizability and stability. Amino acids with small polar (serine, threonine) sidechains were selected for insertion to minimize potential steric clashes with SFTSV Gn and to increase the solubility. Glycine was also inserted to increase the flexibility. A total of 4 peptides (HKU-P1 to HKU-P4) were designed and synthesized for experimental test.

### 2.2. Conformational Modeling of Peptide HKU-P1 Bound to SFTSV Gn

The conformational space of peptide HKU-P1 was searched with Simple Cyclic Peptide Prediction in Rosetta [22]. A total of 10,000 structures were generated. Peptides succeeded in cyclization were superimposed to the native linear pentapeptide to build the initial structure for peptide-SFTSV Gn docking. Rosetta full-atom high-resolution docking protocol was utilized for docking with no repack to both peptide and SFTSV Gn protein. The best pose was selected according to the docking score, hydrogen bonding potential, and root-mean-square deviation (RMSD) to the native peptide structure.

### 2.3. Virus, Cell Lines, and Peptides

SFTSV HB29 strain was kindly provided by Dr. Benjamin Brennan, MRC-University of Glasgow Centre for Virus Research, and Professor Mifang Liang, China CDC. The virus was propagated in Vero cells and kept at −80 °C in aliquots until use. SFTSV was cultured in Vero cells and titrated with plaque formation assay as previously described [14]. African green monkey (Vero) and human hepatoma (Huh-7) cell lines were obtained from American Type Culture Collection and JCRB cell bank of Okayama University, Japan, respectively [23]. Vero and Huh-7 cells were maintained in Dulbecco’s Modified Eagle Medium (DMEM; Thermo Fisher Scientific, Waltham, Massachusetts, USA) with 10% fetal bovine serum (FBS; Thermo Fisher Scientific) as previously described [24]. The peptides were synthesized by Cellmano Biotech Limited (Hefei, Anhui, China). All peptides were assembled on Peptide Synthesizer SyroI (Biotage, Uppsala, Sweden) using the standard solid-phase peptide synthesis method as previously described [25,26]. The peptides were aliquot and stored in −80 °C until use. To check their structural stability, analysis of each peptide using high performance liquid chromatography–mass spectrometry (HPLC-MS) was performed every three months to confirm the absence of peptide degradation. Stock peptide solutions were prepared by dissolving lyophilized peptide in phosphate buffered saline (PBS) to 10 mg/mL. Favipiravir (MedChemExpress, NJ, USA) stock solution was prepared by dissolving in dimethyl sulfoxide (DMSO) to 60 mg/mL. A non-relevant peptide with the amino acid sequence of TRPATLNRFSALQQTL was used as a negative control peptide in this study [27]. Both peptide and favipiravir stock solutions were stored at −80 °C until use. The half-maximal cytotoxic concentration (CC_50_) of peptides in Huh-7 cells were determined by Cell-Titer-Glo^®^ luminescent assay (Promega, Madison, Winconsin, United States) as previously described [14,28]. All experiments involving SFTSV was conducted following the approved standard operation procedures of the Biosafety Level 3 (BSL-3) facility at the Department of Microbiology, The University of Hong Kong.

### 2.4. Expression and Purification of SFTSV Gn Using a Baculovirus Insect Cell Expression System

Recombinant Gn (residues 21–452) of the SFTSV membrane glycoprotein polyprotein from the reference sequence severe fever with thrombocytopenia syndrome virus (GenBank accession no. AZZ69059.1) was expressed and purified in insect cells as we described previously with modifications [29]. Briefly, Gn gene sequences were baculovirus-codon-optimized and cloned into pFast dual baculovirus expression vector. The construct was fused with an N-terminal gp67 signal peptide and C-terminal 6ⅹHis tag for secretion and purification. A recombinant bacmid DNA was generated using the Bac-to-Bac system (Thermo Fisher Scientific). To generate baculovirus carrying the SFTSV Gn, we transfected purified bacmid DNA into Sf9 cells using Cellfectin (Thermo Fisher Scientific) and subsequently used the supernatant to infect ExpiSf9 cell suspension culture (Thermo fisher Scientific) at a multiplicity of infection (MOI) of 1.00 to 10.00. Infected ExpiSf9 cells were incubated at 27.5  °C with shaking at 125 r.p.m. for 72 to 96 h for protein expression. The supernatant was collected and then concentrated using a 30 kDa MW cutoff Labscale TFF System (MilliporeSigma, Burlington, MA, USA). The SFTSV Gn protein was purified by Ni-NTA purification system, followed by size exclusion chromatography, and buffer exchanged into 1ⅹ PBS pH 7.4. The concentration of the purified SFTSV Gn was determined by using the Bradford Assay Kit (Bio-Rad, Hercules, CA, USA) according to the manufacturer’s instructions. The purity of recombinant SFTSV Gn was verified by SDS-PAGE and Western blotting.

### 2.5. Peptide Competition Enzyme-Linked Immunosorbent Assay (ELISA)

Biotinylated HKU-P1 (biotin-HKU-P1) was synthesized by linking biotin to the first lysine residue of HKU-P1 which does not disrupt the cyclic structure. SFTSV Gn protein was expressed and purified as previously described with modifications. A total of 100 ng/well of recombinant SFTSV Gn protein was coated in 96-well ELISA plates for overnight incubation at 4 °C, followed by blocking with 5% bovine serum albumin (BSA) in PBS with Tween-20 (Sigma-Aldrich, St. Louis, MO, USA) for 2 h at 37 °C. All subsequent washing steps were performed with 0.05% Tween-20 in PBS. After washing, biotin- HKU-P1 in PBS was added to the ELISA plate started from the concentration of 1 mg/mL. After 1 h incubation at 37 °C, the plate was washed, horseradish peroxidase (HRP)-conjugated streptavidin (1:1500) was added, and the plate was incubated for 1h at 37 °C. Biotin- HKU-P1 was then detected using 3,3′,3,3′-tetramethylbenzidine (TMB) solution and stop solution (0.1 M HCl). The optical density of each well was read at 450 nm (OD450) using VICTOR3^TM^ multilabel plate reader (PerkinElmer, Inc., Waltham, MA, USA). To evaluate the competition kinetics between modified peptides and biotin-HKU-P1, SFTSV Gn-coated ELISA plates were first incubated with HKU-P1 for 1 h at 37 °C starting at the concentration of 1 mg/mL. Biotin-HKU-P1 at 500 µg/mL was then added. Binding of biotin-HKU-P1 was detected as described above.

### 2.6. Viral Load Reduction Assay

Viral load reduction assay was performed to evaluate the antiviral activity of synthesized peptide against SFTSV as we described previously [14]. Briefly, SFTSV was incubated with peptide starting at 1 mg/mL at 37 °C for 1 h. Huh-7 cells were then infected with SFTSV-peptide complex (MOI = 0.01). At 1-day post-infection (dpi), virus inoculum was removed. The cells were washed with PBS thrice. The viral load in the SFTSV-infected Huh-7 cells was then quantified as previously described with slight modifications [14]. The intracellular viral RNA was extracted using RNeasy Mini Kit (Qiagen, Venlo, Netherlands). The viral load was obtained with reverse transcription quantitative polymerase chain reaction (RT-qPCR) using Quantinova SYBR green PCR kit (Qiagen) using previously established primers for SFTSV (Forward: 5′-GGGTCCCTGAAGGAGTTGTAAA-3′, Reverse: 5′-TGCCTTCACCAAGACTATCAAT GT -3′).

### 2.7. Time-of-Drug-Addition Assay

Time-of-drug-addition (TOA) assay was performed to observe the part of SFTSV replication cycle interfered by the peptides. For the pre-infection group, Huh-7 cells were pre-incubated with HKU-P1 at the concentration of 1 mg/mL at 37 °C for 1 h before infection. For the neutralization group, SFTSV (MOI = 0.50) and HKU-P1 were mixed for 1 h during infecting the Huh-7 cells. At 1 h post-infection (hpi), the virus inoculum was removed, and the SFTSV-infected cells were washed with PBS twice. For the post-infection group, HKU-P1 was added to the SFTSV-infected Huh-7 cells at 1 hpi. The infectious medium was removed at 6 hpi, the Huh-7 cells were washed with PBS thrice, and the cell lysate was collected for viral load measurement.

### 2.8. Immunofluorescence Staining

Huh-7 cells were seeded at 4 × 10^4^ cells/well in NuncTM Lab-TekTm 8-well chamber slide (Thermo Fisher Scientific) one day before infection. SFTSV (MOI = 0.05) was pre-incubated with HKU-P1 at the concentration of 1 mg/mL. The slides were fixed at 24 hpi with 10% formalin (BDH, Merck) in phosphate-buffered saline (PBS) for 15 min. The fixed cells were permeabilized with 0.1% Triton X-100 (Sigma-Aldrich) in PBS and blocked with 5% bovine serum albumin (BSA, Sigma-Aldrich) in PBS. The cells were then stained with in-house mouse anti-SFTSV-NP monoclonal antibody (1:2000 dilution with 1% BSA/PBS) and subsequently Alexa488-conjugated goat anti-mice IgG secondary antibody (Thermo Fisher Scientific) (1:2000 dilution with 1% BSA/PBS). The slides were washed thrice with 0.05% Tween-20 (Sigma-Aldrich) in PBS following each antibody incubation steps. The slides were then mounted with coverslips using VECTASHIELD^®^ Antifade Mountant with 4′,6-diamidino-2-phenylindole (DAPI) (Vector Laboratories, Burlingame, California, USA). All slides were examined, and the images were captured with Olympus BX53 semi-motorized fluorescence microscope using cellSens imaging software as previously described [30].

### 2.9. Drug Combination Assay

Drug combination assay was performed to detect synergistic effects between HKU-P1 and favipiravir. Briefly, SFTSV (MOI = 0.01) was pre-incubated with various concentrations of HKU-P1 (0 to 250 µg/mL) at 37 °C for 1 h. The SFTSV-peptide complex was then mixed with favipiravir, starting at the concentration of 25 µg/mL, before infecting Huh-7 cells. The cells were then washed and lysed for evaluation of intracellular viral RNA load at 1 dpi. The Loewe additivity index was calculated as previously described [31].

## 3. Results

### 3.1. Peptide Design on the Basis of Hotspot Residues of MAb 4-5 and SFTSV Gn Binding

**Figure 1:**
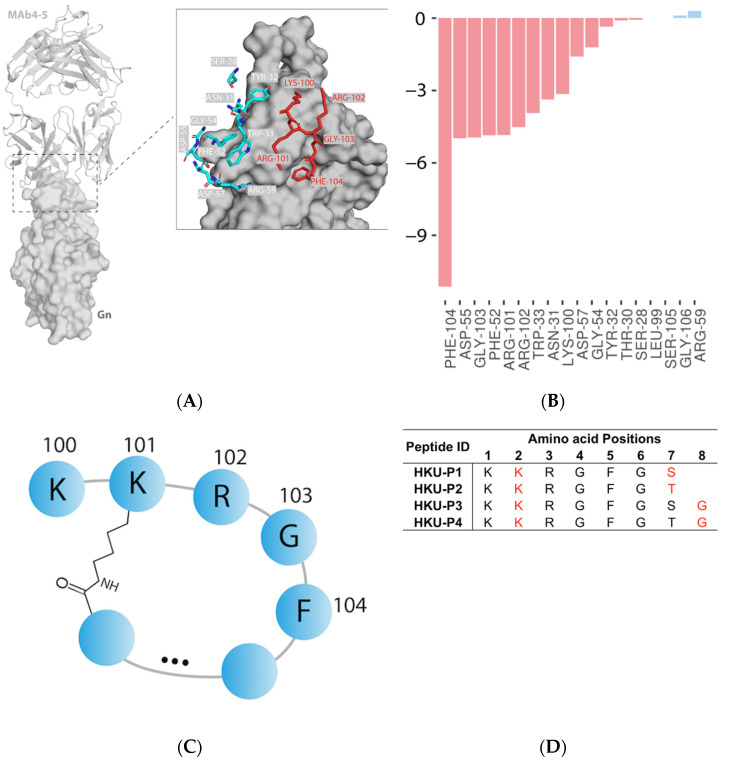
Antibody-based cyclic peptide design strategy. (**A**) Structural representation of MAb 4-5 (cartoon) and SFTSV Gn (surface) complex (PDB code: 5Y11). Interface residues of MAb 4-5 within 4.0 Å of SFTSV Gn in protein–protein interaction are represented by sticks. The pentapeptide selected for extension and cyclization are highlighted in red. (**B**) Residue-wise energy breakdown of the SFTSV Gn-MAb binding interface. Attractive and repulsive energies are shown in red and blue, respectively. (**C**) Diagram of the pentapeptide extension and cyclization. (**D**) The amino acid sequences of the four newly designed cyclic lariat peptides. The isopeptide bond-forming residues are highlighted in red.

Our study is based on a previously identified SFTSV Gn neutralizing antibody, designated MAb 4-5. The complex structure of the SFTSV Gn head and MAb 4-5 reveals that helice α6 in subdomain III is the key component for neutralization [16]. At the interface, a total of 14 MAb 4-5 residues within 4.0 Å of the SFTSV Gn surface were involved in the protein–protein interaction (Figure 1A). From the interface score calculation, the ΔG of MAb 4-5 and SFTSV Gn binding is roughly −48.7 Rosetta Energy Units (REU). After residue-wise energy breakdown, PHE^104^ was revealed to contribute the most (>20% of the total energy) to the binding free energy (−11.1 REU) (Figure 1B). Residue PHE^104^ was therefore designated as an anchor of peptide design due to its strong nonpolar contacts with a small SFTSV Gn surface cavity. To this end, a pentapeptide consisting of ARG^101^, LYS^100^, ARG^102^, GLY^103^, and PHE^104^ was selected for extension and cyclization by forming an isopeptide bond to ARG-102 sidechain (Figure 1C). ARG-102 was substituted to lysine for the sake of similar sidechain structure and better synthesizability and stability. Finally, 4 cyclic peptides (HKU-P1 to HKU-P4) were designed and synthesized for downstream experiments (Figure 1D).

### 3.2. Evaluation of the Anti-SFTSV Activity of the Cyclized Peptides

**Figure 2:**
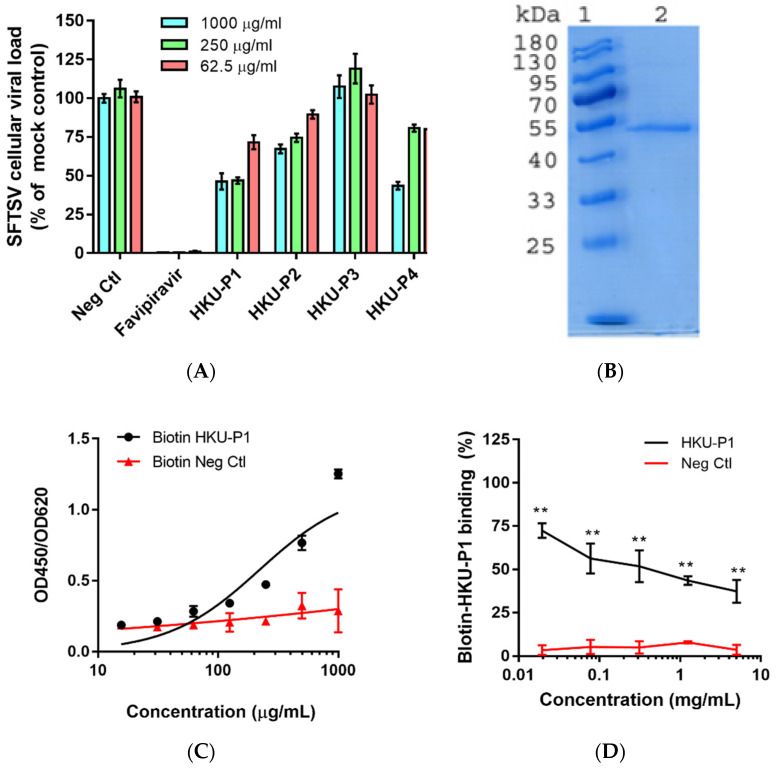
Evaluation of the cyclic peptides’ anti-SFTSV activity and HKU-P1′s binding competition with the biotinylated cyclic peptide. (**A**) Anti-SFTSV activity of cyclic peptides HKU-P1 to HKU-P4. Out of the four peptides, HKU-P1, HKU-P2, and HKU-P4 displayed anti-SFTSV effects. (**B**) Sodium dodecyl sulfate-polyacrylamide gel electrophoresis showing the purity of His-tagged SFTSV Gn (lane 2). Lane 1 represents protein molecular weight marker. (**C**) Detection of the biotin-HKU-P1 binding to SFTSV Gn-coated immunoplate. (**D**) HKU-P1 inhibited the binding of biotin-HKU-P1 to the SFTSV Gn protein. ** *p* < 0.01 when compared the HKU-P1 and negative control peptide at each concentration by Student’s *T*-test.

SFTSV was incubated with peptides at 1000 µg/mL at 37 °C for 1h before infecting Huh-7 cells (multiplicity of infection (MOI) = 0.05). Favipiravir was included as a positive control inhibitor, and a non-relevant scramble peptide was taken as a negative control [27]. At 1 day post-infection (dpi), the virus inoculum was removed, and the intracellular SFTSV viral RNA load was determined. Among the 4 cyclic peptides, peptide HKU-P1 displayed the best anti-SFTSV activity with >50% reduction in SFTSV viral RNA load compared to the mock treatment control group. Peptides HKU-P2 and HKU-P4 also displayed some anti-SFTSV activity with >30% reduction in the intracellular viral RNA load, whereas peptide HKU-P3 exhibited marginal anti-SFTSV effect (Figure 2A). Peptide HKU-P1 was thus subjected to SFTSV Gn protein binding assay.

### 3.3. HKU-P1 Exhibits Binding Activity to SFTSV Gn Protein

ELISA-based binding competition assay was used to confirm the binding of peptide HKU-P1 to the SFTSV Gn protein. The SFTSV Gn protein was expressed (Figure 2B) and purified using baculovirus expression system, and the protein was coated onto ELISA immune plate [29]. We first tested whether biotin-HKU-P1 could bind to the SFTSV Gn protein. ELISA showed that the binding of biotin-HKU-P1 to SFTSV Gn-coated ELISA plate was dose-dependent, showing a binding affinity of 700 µg/mL (Figure 2C). To select the optimal concentration of biotin-HKU-P1 for the competition binding assay, serial dilutions of biotin-HKU-P1 were added to the SFTSV Gn-coated ELISA plate for binding signal detection. Biotin-HKU-P1 at 250 µg/mL exhibited markedly larger (2–3 folds) signal window than the background signal and was thus selected for the subsequent competition binding assay. We then tested peptide HKU-P1 against 250 µg/mL of biotin-HKU-P1 to see if they could compete for the binding to the SFTSV Gn protein. As shown in Figure 2D, HKU-P1 inhibited the binding of biotin-HKU-P1 in a dose-dependent manner. The half-maximal inhibitory concentration (IC_50_) of HKU-P1 was 94.5 µg/mL. These results confirmed that HKU-P1 is an SFTSV Gn binder with antiviral activity.

### 3.4. HKU-P1 Inhibits SFTSV Replication

**Figure 3:**
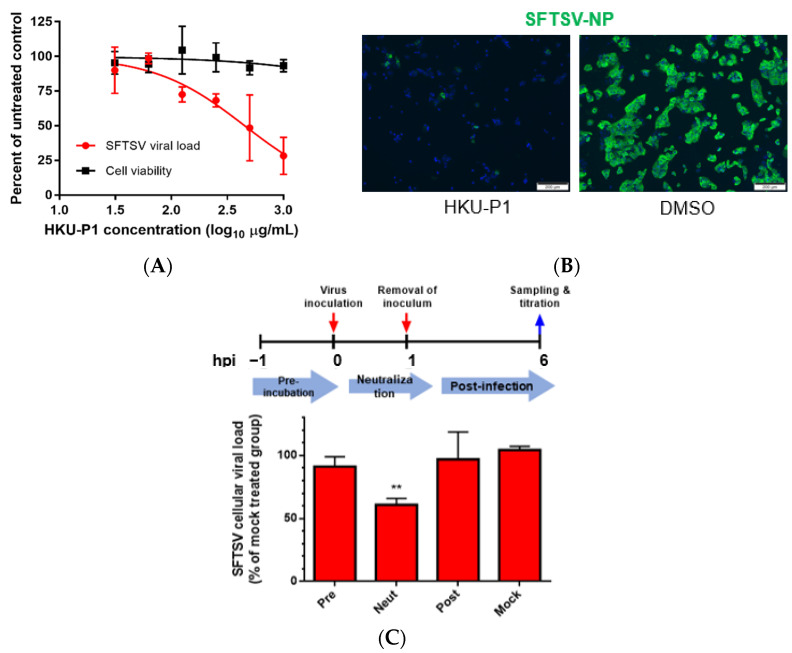
The anti-SFTSV activity and mode of action of cyclic peptide HKU-P1. (**A**) Evaluation of the in vitro anti-SFTSV activity and cell toxicity of HKU-P1. The SFTSV intracellular viral RNA load at 1 day post infection (MOI = 0.01) in Huh-7 cells with HKU-P1 was quantified by RT-qPCR. Cell viability was determined at the same condition using CellTiter-Glo assay. SFTSV viral RNA load was normalized as % of the mock-treated control group. HKU-P1 displayed dose-dependent reduction of the SFTSV viral RNA load with >80% (*p* = 0.010) viral RNA load reduction being achieved at 1000 µg/mL of HKU-P1. The half-maximal inhibitory concentration (IC_50_) was calculated using non-linear regression analysis in GraphPad Prism. (**B**) Immunofluorescence staining of SFTSV-NP in virus-infected Huh-7 cells treated with HKU-P1. (**C**) Time-of-drug-addition assay was performed to determine the step in the SFTSV replication cycle that is interrupted by HKU-P1. HKU-P1 was either pre-incubated with Huh-7 cells at 1h pre-infection (Pre), co-incubated with SFTSV during virus entry (Neut), or added at 1 h post-infection (Post). The viral RNA load was normalized as % of mock-treated control group. Significant viral load reduction was observed in the neutralization group. This was compatible with the postulated mechanism of the cyclic peptides as they were designed as SFTSV-Gn binders. ** denotes *p* < 0.01 (compared to the mock-treated control group with Student’s t-test). Data are presented as mean values ± standard deviations (*n* = 3). All experiments were performed in triplicate and repeated twice for confirmation.

To further characterize the anti-SFTSV activity of peptide HKU-P1, viral load reduction assay was performed. SFTSV was first incubated with HKU-P1 starting at the concentration of 1000 μg/mL. Huh-7 cells were then infected with SFTSV-peptide complex, and intracellular viral RNA load was measured at 1 dpi. HKU-P1 reduced SFTSV viral load in a dose-dependent manner (Figure 3A). At the concentration of 1000 μg/mL, HKU-P1 significantly reduced SFTSV intracellular viral RNA load by >70% (*p* = 0.027). The IC_50_ of HKU-P1 was 523.9 μg/mL. HKU-P1 exhibited negligible cytotoxicity at 1000 μg/mL, and it was impracticable to test for cytotoxicity at higher concentration because the stock concentration was 10 mg/mL. Thus, the CC_50_ should be well above 1000 μg/mL. The anti-SFTSV activity of HKU-P1 was further demonstrated with immunofluorescence staining. SFTSV was incubated with HKU-P1 at the concentration of 1000 μg/mL before infecting Huh-7 cells. The cells were then fixed and stained with monoclonal mouse anti-SFTSV-NP antibody. Immunofluorescence staining showed that SFTSV-NP expression was markedly reduced by HKU-P1 (Figure 3B).

### 3.5. HKU-P1 Neutralizes SFTSV Virion

Given that the cyclic peptide HKU-P1 was derived from anti-SFTSV-Gn monoclonal antibody (MAb 4-5), virus neutralization was the postulated antiviral mechanism of HKU-P1. To determine the step of the viral replication cycle interrupted by HKU-P1, we conducted a time-of-drug-addition (TOA) study in which HKU-P1 was added to Huh-7 cells at the pre-infection stage, co-infection stage, or post-infection stage (Figure 3C). For the pre-infection group, HKU-P1 (1000 μg/mL) was pre-incubated with Huh-7 cells at 1h before infection. For the co-infection/neutralization group, HKU-P1 was pre-incubated with SFTSV for 1h before infection, and no peptide was added to Huh-7 cells at 2 hpi after removal of the initial inoculum. For the post-infection group, HKU-P1 was only added at 2 hpi after the removal of the inoculum. The SFTSV intracellular viral RNA load was then determined at 6 hpi. Compared to the mock treatment group, the SFTSV viral RNA load in the neutralization group was significantly (*p* = 0.0011) reduced by >40% reduction, while there was no significant viral load reduction in the pre-infection group nor post-infection group. This suggested that HKU-P1 was highly likely neutralizing SFTSV virion without interrupting other replication events of the SFTSV life cycle.

### 3.6. Conformational Sampling of HKU-P1 Bound to MAb 4-5 Binding Epitope of SFTSV Gn

**Figure 4:**
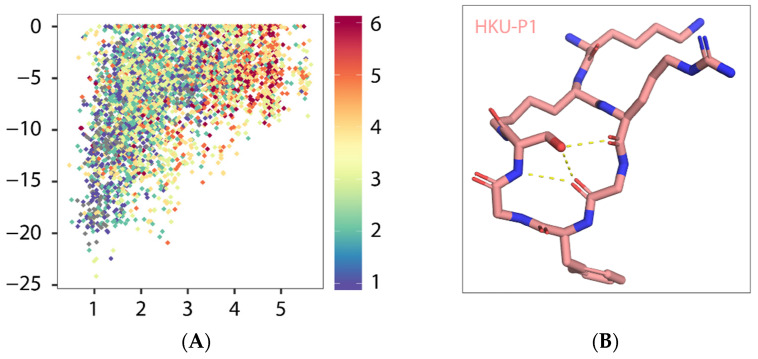

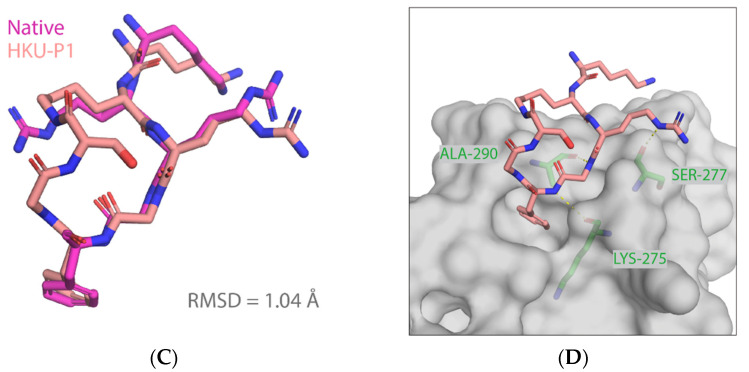
Predicted binding mode of HKU-P1 to SFTSV Gn. (**A**) Conformational landscape analysis of HKU-P1 bound to SFTSV Gn, showing computed binding energy against RMSD to native pentapeptide. Each point represents a conformational sampling attempt succeeded in cyclization. Colors indicate the number of intramolecular hydrogen bonds. (**B**) Intramolecular hydrogen bonding in top conformation of HKU-P1; hydrogen bonds were indicated with yellow dashed lines. (**C**) Overlay of top conformation and native pentapeptide. The RMSD value of aligned part was labelled. (**D**) Binding mode of HKU-P1 top conformation to the SFTSV Gn surface. HKU-P1 top conformation and SFTSV Gn were represented by sticks and surfaces, respectively. Hydrogen bonding residues of SFTSV Gn were shown in green sticks and labelled, and hydrogen bonds were indicated with yellow dashed lines.

HKU-P1 (amino acid sequence: LYS-LYS-ARG-GLY-PHE-GLY-SER) was constructed by appending two residues glycine and serine to PHE^104^ and connected to the sidechain lysyl amide of the second lysine with serine C-termini carboxyl. To characterize the folding propensity to native pentapeptide conformation, HKU-P1 was subjected to a large-scale conformational landscape analysis. Out of the 10,000 cyclization attempts, more than 4000 runs were successful. When docked to the native pentapeptide binding site, a funnel-like shape of the score-versus-RMSD was obtained, where large RMSD values corresponded to poor scores, and as RMSD approaches the native, rapidly improving scores, it leads to a cluster of near-native conformations (Figure 4A). The top conformation of HKU-P1 (binding score −24.2 REU) has three intramolecular backbone hydrogen bonds (Figure 4A,B), suggesting good rigidity in binding-competent conformation, which is a key determinant of high-affinity binding. In addition, the RMSD between top conformation and the native pentapeptide in aligned part was 1.04 Å, indicating that HKU-P1 was able to mimic the native pentapeptide binding (Figure 4C). Apart from the strong nonpolar contacts between HKU-P1 and SFTSV Gn, three intermolecular hydrogen bonds were formed as seen in Figure 4D, which were assumed to further improve the binding affinity and specificity. In conclusion, HKU-P1 binds to Gn in a similar mode with native pentapeptide as designed.

### 3.7. HKU-P1 and Favipiravir Display Synergistic Anti-SFTSV Effect

We further explored the possibility of applying HKU-P1 in combination with preexisting anti-SFTSV drug compounds to produce synergistic antiviral effect. Among the drug compounds evaluated for their antiviral activity against SFTSV, favipiravir, a nucleoside analogue which inhibits viral RNA-dependent RNA polymerase (RdRp), is the only one which has consistently demonstrated in vitro and in vivo activity. Favipiravir displayed broad-spectrum antiviral effects against various RNA viruses, including SFTSV [18]. As favipiravir is likely to inhibit the post-entry replication events in the SFTSV life cycle, it is unlikely to compete with viral neutralization peptides such as HKU-P1. We therefore tested whether peptide HKU-P1 could potentiate the anti-SFTSV activity of favipiravir. SFTSV (MOI = 0.01) was first incubated with HKU-P1, at concentrations below the peptide’s IC_50_ (0 to 250 μg/mL, i.e., subpotent concentrations), for 1h before infecting Huh-7 cells. At 0 hpi, SFTSV-peptide complex and favipiravir were added to Huh-7 cells. Intracellular SFTSV viral RNA load was determined at 1dpi. The IC_50_ of favipiravir at each HKU-P1 concentration was then calculated (Table 1). Favipiravir, when used alone, exhibited an IC_50_ of 1.25 µg/mL. Favipiravir was combined with 250, 125, or 62.5 µg/mL of HKU-P1, and the IC_50_ of favipiravir decreased to 0.805 µg/mL, 0.864 µg/mL, and 0.979 µg/mL, respectively. In the Loewe additivity index (>1: antagonism; =1: additive; <1: synergism) the drug combinations ranged from 0.56 to 0.60 (Table 1) [31], indicating synergistic effects between HKU-P1 and favipiravir.

## 4. Discussion

Peptide and peptide-based inhibitors represent an attractive alternative to small molecule compounds because peptides may be more specific for blocking the interface between large protein–protein interaction sites and better tolerated than small molecule compounds [32]. Clinical application of the anti-HIV peptide enfuvirtide demonstrates that peptidic antivirals can be a safe and effective alternative for the treatment of infectious diseases [33,34]. However, due to their relatively large sizes and susceptibilities to proteolytic degradation, peptides may have relatively low oral bioavailabilities and short half-lives. These suboptimal ADME (absorption, distribution, metabolism, elimination) properties often limit their development for clinical use [35]. With the realization that macrocyclization can dramatically improve the binding affinity and proteolytic stability of peptides, there have been increasing efforts to find novel means to cyclize peptides [36]. In this study, we demonstrated the design and antiviral efficacy of a novel cyclic peptide HKU-P1 against the highly virulent SFTSV.

In recent years, many peptides with antiviral activity in vitro have been identified using a variety of methods, such as structure-based identification [37] and brute force trial and error [38]. For example, antiviral peptides have been identified by scanning the sequence of viral fusion proteins (glycoproteins) for sequences that have membrane binding potential, determined by the Wimley–White interfacial hydrophobicity scale (WWIHS) [39,40]. Using this method, antiviral peptides for various emerging viruses such as influenza viruses and coronaviruses have been generated [41,42,43]. In this study, on the basis of Rosetta suite, we developed a robust pipeline for the design of antiviral peptides. Rosetta is a powerful software that allows accurate design of a novel protein structure, prediction of the structure of protein−protein complexes, and design of protein−protein and protein−DNA interactions [44]. Our study indicates that this platform may also facilitate the design of novel antiviral peptides for other emerging viruses with resolved crystal structures.

In addition to its antiviral activity against SFTSV, it would be interesting to evaluate the antiviral activity of HKU-P1 against other members of the genus *Bandavirus* in the order *Bunyavirales*. For example, the medically important Heartland virus (HRTV) that was first isolated from patients in the United States in 2012 is closely related to SFTSV [45]. The M segment of the two viruses is 61.9% amino acid, and they are identical to each other [45]. K288 (based on SFTSV numbering) of the SFTSV Gn is the key residue involved in HKU-P1 binding. The corresponding residue in HRTV is arginine, which is also a positively charged amino acid, suggesting that HKU-P1 may also bind with the HRTV Gn and neutralize the virus. Therefore, the potential of HKU-P1 as a broad-spectrum antiviral peptide against different bandaviruses should be further investigated in future studies.

As SFTSV causes severe disease with a high mortality rate, combinatorial treatment with multiple antivirals targeting different steps of the viral replication cycle may be a preferred strategy over monotherapy. Favipiravir is a broad-spectrum viral RdRp that has previously been shown to exhibit in vitro and in vivo activity against SFTSV [17,18]. However, as favipiravir is not readily available in most countries, it would be desirable to develop additional antivirals that possess synergistic or additive effects with favipiravir in order to minimize the dosage of favipiravir required. Our results showed that HKU-P1 is consistently synergistic with favipiravir at different concentrations (Loewe additivity index < 1).

## 5. Conclusions

In summary, the novel cyclic peptide HKU-P1 potently neutralizes SFTSV. The anti-SFTSV effect of combinatorial HKU-P1 and favipiravir should be further evaluated in suitable animal models such as the interferon receptor-deficient A129 mouse model.

## Figures and Tables

**Table 1:** Effect of combinatorial HKU-P1 and favipiravir on SFTSV replication. The favipiravir IC_50_ at each concentration of HKU-P1 was calculated using non-linear regression analysis in GraphPad Prism. Loewe additivity index represents the drug relationship (>1: antagonistic; =1: addition; <1: synergistic).

		Favipiravir IC_50_ (µg/mL)	Loewe Additivity Index
HKU-P1 concentration (μg/mL)	250	0.805 ± 0.105	0.60
	125	0.864 ± 0.132	0.56
	62.5	0.979 ± 0.194	0.59
	0	1.247 ± 0.101	-

## Data Availability

All data can be found in this manuscript.
